# Supplementary material for: Retinoic acid rewires the adrenergic core regulatory circuitry of childhood neuroblastoma
Source: Sci Adv. 2021 Oct 20;7(43):eabe0834. doi: 10.1126/sciadv.abe0834 (PMC8528416; doi:10.1126/sciadv.abe0834)
Supplement: Supplementary file 1 — Figs. S1 to S8 Tables S1 to S4 [file sciadv.abe0834_sm.pdf]

Science Advances
AAAS

Supplementary Materials for

Retinoic acid rewires the adrenergic core regulatory circuitry of childhood neuroblastoma

Mark W. Zimmerman*, Adam D. Durbin, Shuning He, Felix Oppel, Hui Shi, Ting Tao, Zhaodong Li, Alla Berezovskaya, Yu Liu, Jinghui Zhang, Richard A. Young, Brian J. Abraham*, A. Thomas Look*

*Corresponding author. Email: markw_zimmerman@dfci.harvard.edu (M.W.Z.); brian.abraham@stjude.org (B.J.A.); thomas_look@dfci.harvard.edu (A.T.L.)

Published 20 October 2021, *Sci. Adv.* **7**, eabe0834 (2021)
DOI: 10.1126/sciadv.abe0834

This PDF file includes:

Figs. S1 to S8
Tables S1 to S4

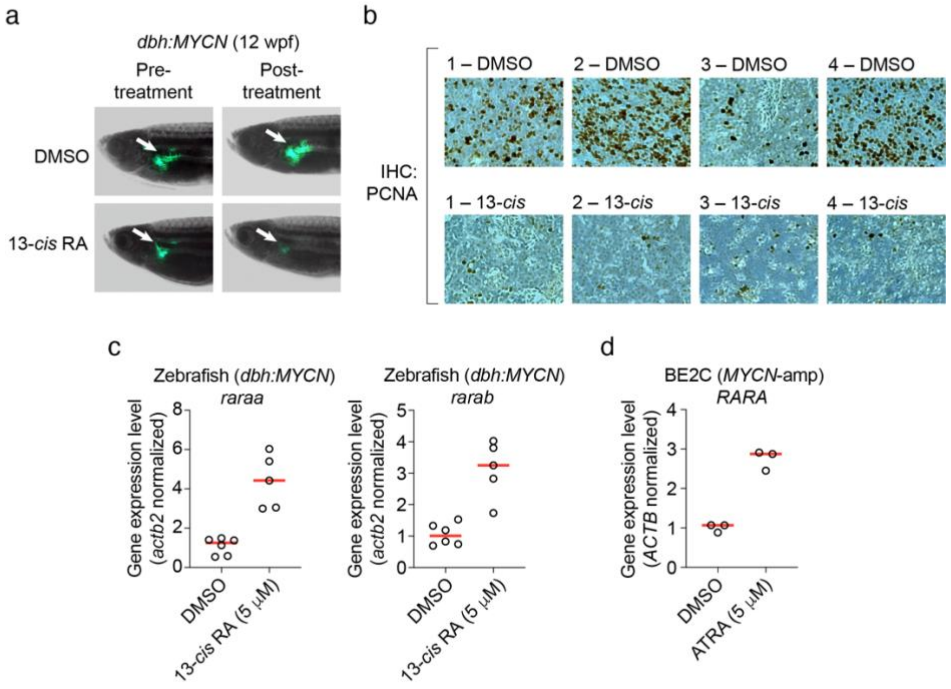

Figure S1. Loss of neuroblastoma cell proliferation following treatment with 13-*cis* retinoic acid.

a) Representative images of 12 week-old *dbh:MYCN* transgenic zebrafish with EGFP+ neuroblastomas (white arrows) before and after treatment with DMSO or 5 μM 13-*cis* retinoic acid for 6 days. b) Images of individual 12 wpf neuroblastoma tumor sections treated with DMSO (above) or 5 μM 13-*cis* retinoic acid (below) for 6 days and stained with a PCNA detecting antibody counter stained with hematoxylin. c) Relative gene expression levels of *raraa* and *rarab* assayed by quantitative RT-PCR in adult zebrafish tumors treated with DMSO or 5 μM 13-*cis* retinoic acid for 6 days. d) Relative gene expression levels of *RARA* assayed by quantitative RT-PCR in *MYCN*-amplified BE2C cells treated with DMSO or 5 μM ATRA for 6 days.

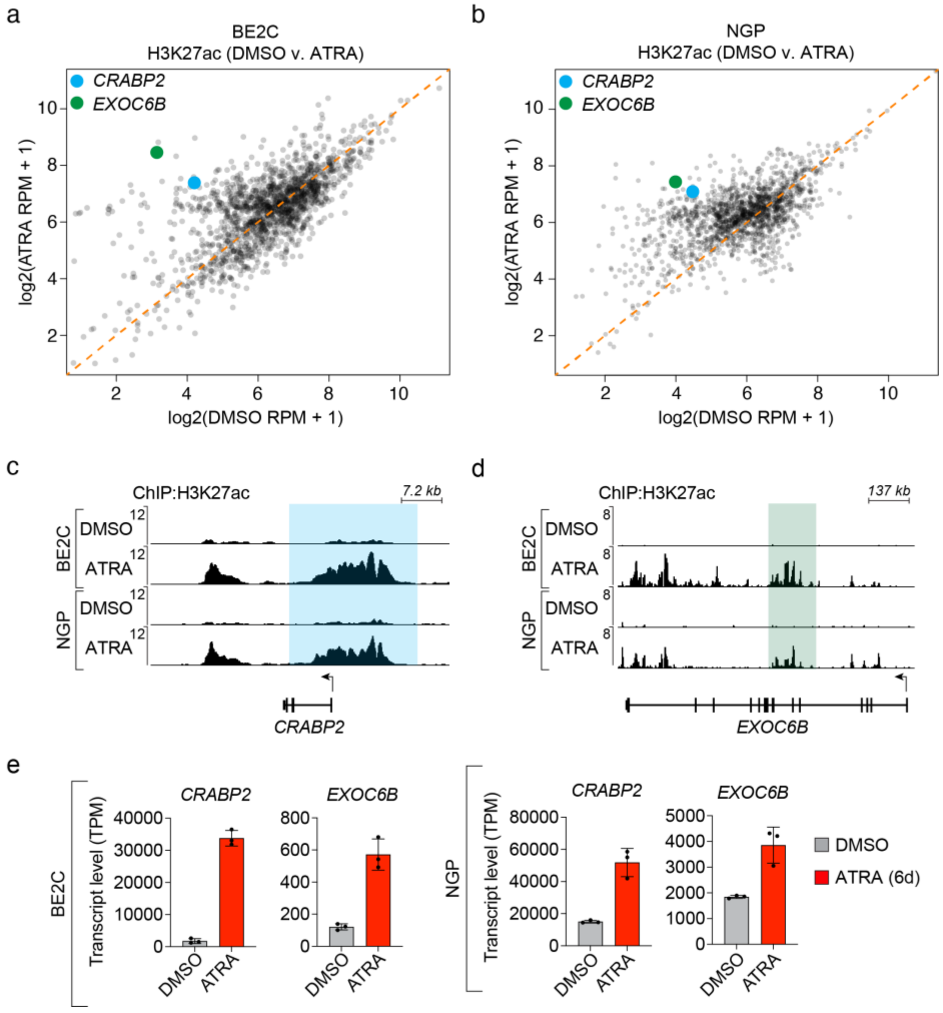

Figure S2. Acquisition of new super-enhancers is associated with increased expression of their associated genes.

a,b) Super-enhancers were identified in the DMSO and ATRA treated BE2C (a) and NGP (b) cells and collapsed into one set of regions whose differential enrichment was assessed in a H3K27ac coverage scatterplot. Orange diagonal line indicates enhancers whose H3K27ac signal was equal in for DMSO compared to ATRA treatment. Highlighted enhancers were associated with *CRABP2* (light blue) and *EXOC6B* (green). c,d) Normalized ChIP-seq tracks for H3K27ac showing acquired super-enhancers associated with the *CRABP2* (c) and *EXOC6B* (d) gene loci in BE2C and NGP cells. Cells were treated with 5 μM ATRA for 12 days; shaded areas indicate super-enhancers called by H3K27ac in ATRA-treated cells. ChIP-seq read densities (y axis) were normalized to reads per million reads sequenced from each sample. e) Expression levels of *CRABP2* and *EXOC6B* determined by spike-in normalized mRNA-seq in BE2C and NGP cells treated with DMSO (grey) or 5 μM ATRA (red) for 6 days.

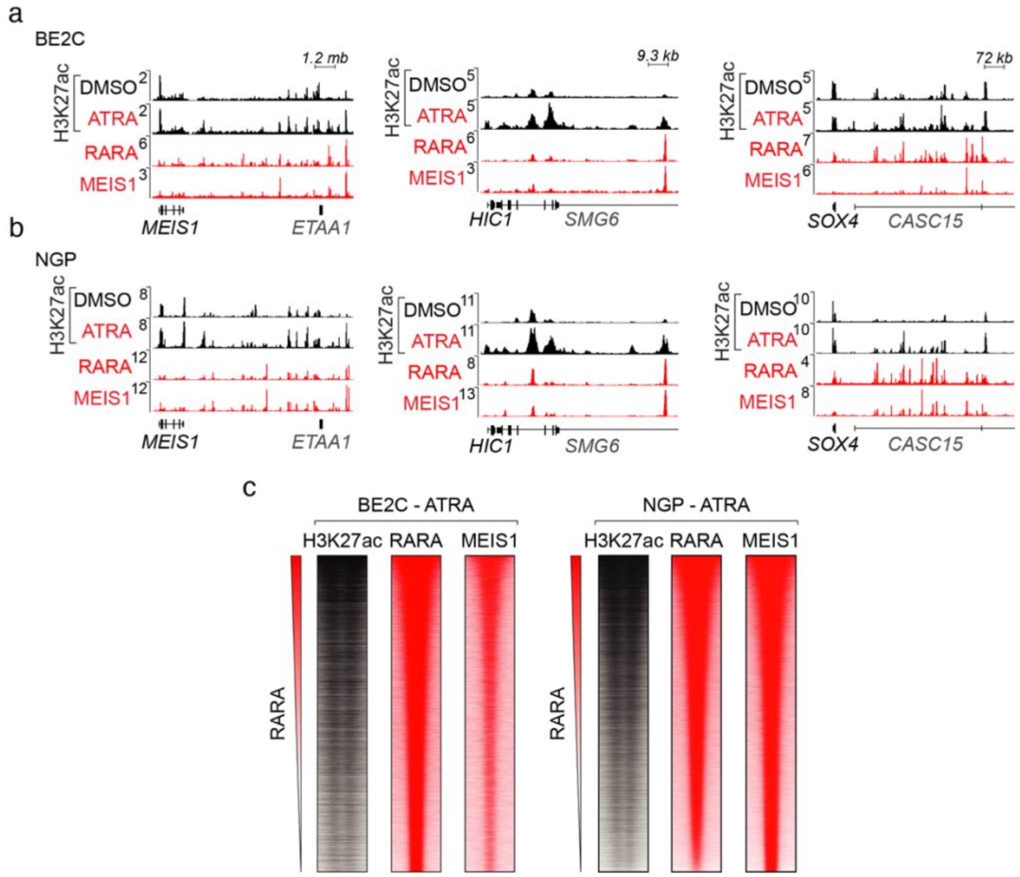

Figure S3. RARA and MEIS1 occupy H3K27ac-enriched super-enhancers associated with *MEIS1*, *HIC1* and *SOX4* in ATRA-treated neuroblastoma cells. a-b) Normalized ChIP-seq tracks depicting H3K27ac in DMSO- and ATRA-treated (12 days) BE2C (a) and NGP (b) cells, and ChIP-seq for RARA and MEIS1 in ATRA-treated cells showing the regions surrounding the *MEIS1* (left), *HIC1* (center) and *SOX4* (right) gene loci. ChIP-seq read densities (y axis) were normalized to reads per million reads sequenced. c) Genome-wide co-occupancy for H3K27ac, RARA and MEIS1 in ATRA-treated BE2C and NGP cells as determined by ChIP-seq. Genomic regions (rows) were defined as those enriched in sequencing reads for at least one target and are ranked by the RARA signal therein.

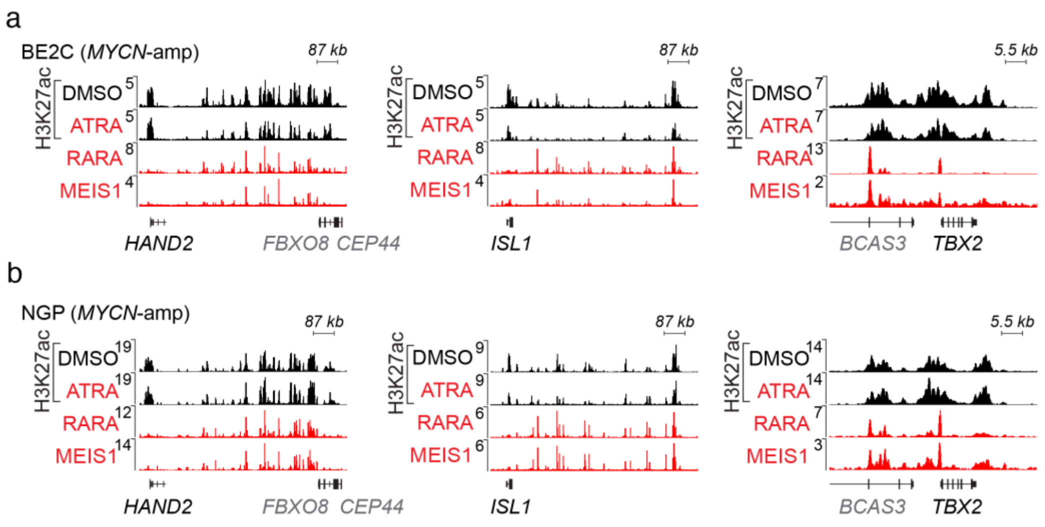

Figure S4. Several super-enhancers associated with CRC transcription factors are stable after treatment with ATRA. a-b) Normalized ChIP-seq tracks depicting H3K27ac distribution in 12-day DMSO- and ATRA-treated BE2C (a) and NGP (b) cells, and ChIP-seq for RARA and MEIS1 in ATRA-treated cells showing the regions surrounding the *HAND2* (left), *ISL1* (center) and *TBX2* (right) gene loci. ChIP-seq read densities (y axis) were normalized to reads per million reads sequenced.

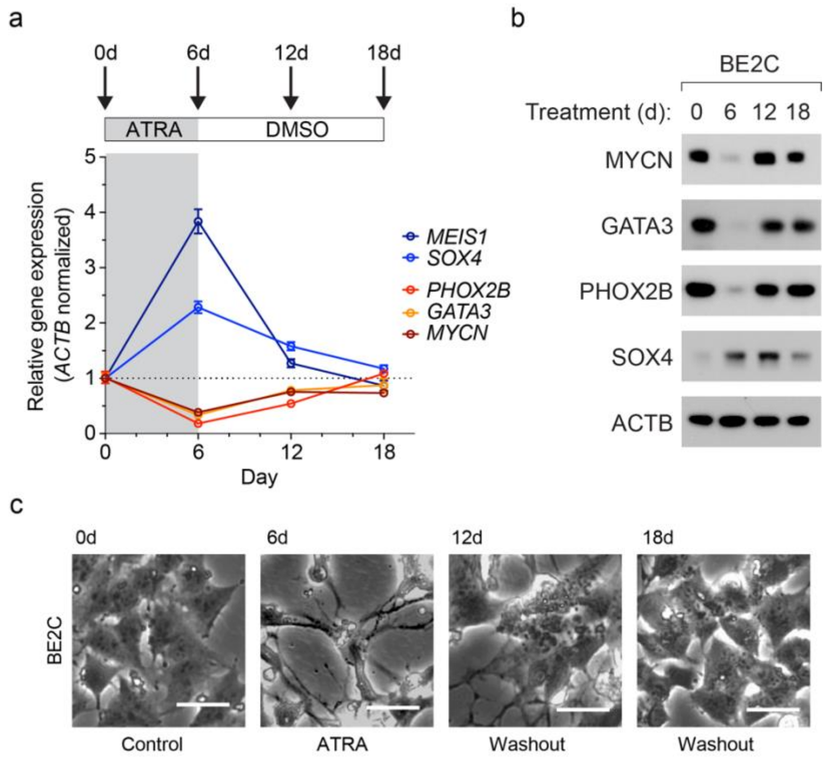

Figure S5. Reversion to the adrenergic CRC and phenotype following ATRA washout.

a) *MYCN*-amplified BE2C cells treated with 5 μM ATRA for 6 days followed by washout and culturing for 12 days and relative gene expression levels of *MEIS1*, *SOX4*, *PHOX2B*, *GATA3* and *MYCN* were assayed by quantitative RT-PCR at 0, 6, 12 and 18 days. Expression levels were normalized to *ACTB* levels and plotted relative to pretreatment values at each time point. b) In BE2C cells were treated with 5 μM ATRA for 6 days, protein levels of MEIS1, SOX4, PHOX2B, GATA3 and MYCN were assayed by Western blotting before and after 6 days of ATRA treatment and then at 6 and 12 days after washout of ATRA. c) Representative brightfield images of BE2C cells treated with 5 μM ATRA for 6 days and imaged at 0 (pre) and 6 days (during ATRA treatment). Cells were also imaged at 6 and 12 days after washout. Scale bar indicates 10 μm.

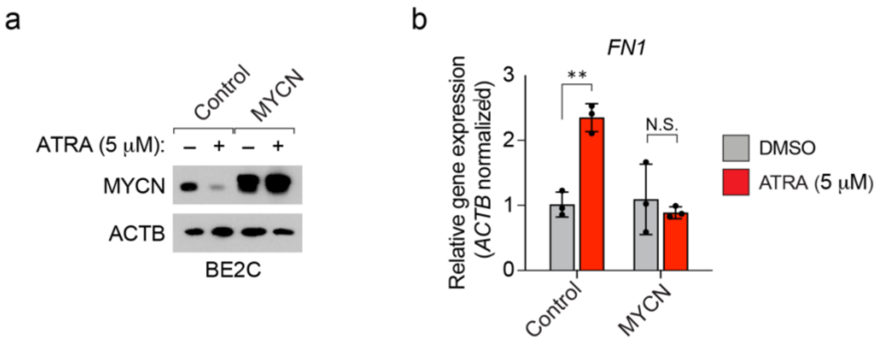

Figure S6. Retained expression of MYCN blocks the induction of the ATRA-mediated differentiation program in neuroblastoma.

a) Western blot assay for MYCN protein levels in control and MYCN-transduced BE2C cells treated with 5 μM ATRA for 48 hr. ACTB is used as a loading control. b) Expression of *FN1* RNA was assayed by quantitative RT-PCR normalized to *ACTB* in control and MYCN transduced BE2C cells treated with 5 μM ATRA for 48 hr, **$p<0.01$ by T-test; not significant (N.S.).

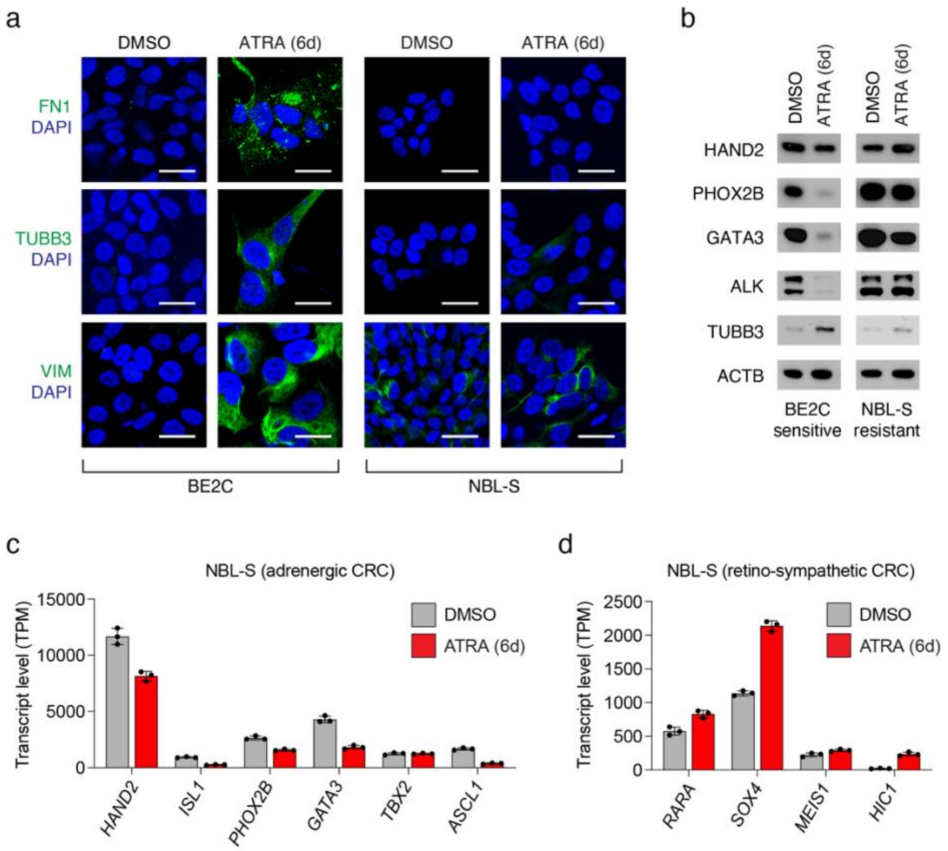

Figure S7. ATRA-mediated changes in gene expression and protein level in *MYCN*-amplified cells are not observed in cells that activate *MYCN* or *MYC* by enhancer hijacking. a) Confocal images of BE2C (*MYCN*-amplified) and NBL-S (*MYCN* enhancer hijacking) neuroblastoma cells treated with DMSO or 5 μM all-*trans* retinoic acid (ATRA) for 6 days. Cells were stained with fibronectin (FN1), b3-tubulin (TUBB3) or vimentin (VIM) (green) and counterstained with DAPI (blue). Scale bar indicates 25 μm. b) Western blot assay for CRC transcription factors (HAND2, PHOX2B and GATA3), ALK (ATRA-suppressed) and TUBB3 (ATRA-upregulated) in BE2C (sensitive) and NBL-S (resistant) cells treated with DMSO or 5 mμM ATRA for 6 days. ACTB is the protein loading control. c) Transcript levels determined by spike-in normalized RNA-seq for adrenergic CRC dependency genes (left) and retino-sympathetic CRC genes (right). NBL-S (resistant) cells exhibit partial upregulation of the retino-sympathetic CRC, but do not sufficiently suppress the *PHOX2B* and *GATA3* adrenergic CRC components.

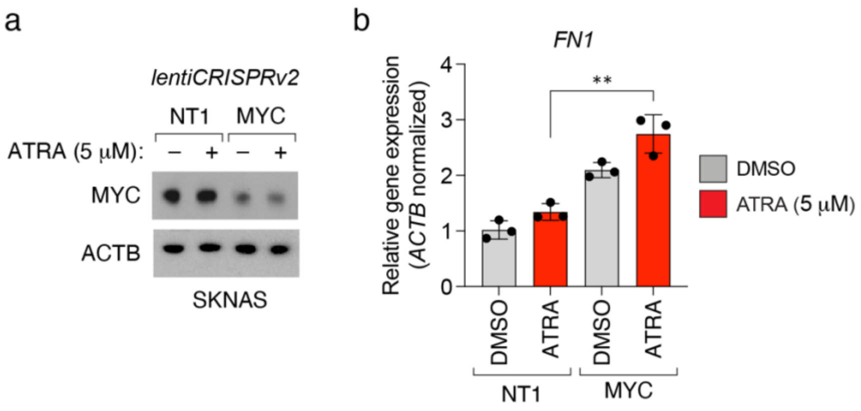

Figure S8. Disruption of *MYC* activity sensitizes cells to the transcriptional effects of ATRA. a) Western blot showing loss of MYC protein in SKNAS *MYC*-enhancer hijacked cells that were transduced with lentiviral constructs expressing Cas9 a sgRNA targeting *MYC* or a control non-targeting sgRNA (NT1). Treatment with ATRA for 2 days did not affect the MYC protein level in either control or MYC-disrupted SKNAS cells. b) Quantitative RT-PCR assay for expression of the retino-sympathetic downstream gene *FN1*. Unlike control cells that do not significantly upregulate *FN1* in response to ATRA, MYC-depleted cells show elevated expression of *FN1*, which is further elevated in response to ATRA (**p<0.005).

Adrenergic only		Retino-sympathetic only		Adrenergic and retino-sympathetic	
**BE2C (*MYCN*-amp)**	**NGP (*MYCN*-amp)**	**BE2C (*MYCN*-amp)**	**NGP (*MYCN*-amp)**	**BE2C (*MYCN*-amp)**	**NGP (*MYCN*-amp)**
GATA2	ID3	SOX4	SOX4	HIF1A	MLXIP
TUB	UBTF	RARA	MEIS1	HAND2	PBX1
INSM2	INSM2	HEY1	ZFHX3	TBX2	HAND2
HAND1	GATA3	JUNB	ETV1	TBX3	SOX11
GATA3	PHOX2A	ETS1	RARA	NFE2L1	TBX3
GATA4	TCF4	FOXO3	RARB	TFAP2B	MEIS2
PHOX2B	PHOX2B	MEIS1	TSHZ3	KLF7	TBX2
NFATC4	HAND1		KLF12	TCF4	NR2F1
ID3	GATA2		ZEB2	TEAD1	ARID1A
ZNF217	SOX12		MEF2D	TCF12	TEAD1
TCF7L2	RCOR2		ETS1	MEIS2	NFE2L1
	NEUROD1		HIC1	NFIB	RERE
	PROX1		PBX3	KLF13	TUB
	ISL1		FOXO3	ZFHX3	NFIB
	TLX2		NFATC4	FEV	TWIST1
			JUND	ISL1	KLF13
			HLX	ID2	ID2
				PROX1	ARID1B
				MLXIP	KLF7
				ARID1B	ZBTB40
				ESRRG	CREB1
				PBX3	
				ETV1	
				TWIST1	

Table S1. Putative core regulatory transcription factors: List of genes identified as putative core regulatory transcription factors in BE2C and NGP cells based on three criteria: 1) associated with super-enhancers, 2) high gene expression level (top 10% of expressed transcripts), and 3) classified as a transcription factor. Genes meeting these criteria in both the adrenergic (DMSO-treated) and retino-sympathetic (ATRA-treated) cell states are listed to the right, and genes unique to the adrenergic and retino-sympathetic cell states are listed to the left. The adrenergic, retino-sympathetic, and shared CRC transcription factors common to both BE2C and NGP cells are highlighted in red.

PCR primer	Sequence (5'–3')
MYCN-For	CACAGTGACCACGTCGATTT
MYCN-Rev	CACAAGGCCCTCAGTACCTC
HAND2-For	CAGCAACGACAAGAAAACCA
HAND2-Rev	GGATGATTCCAAATGCAAGG
ISL1-For	GGCATGTTTGAAATGTGCGG
ISL1-Rev	ACACAGCGGAAACACTCGAT
PHOX2B-For	CTTCGCGGAGACTCACTACC
PHOX2B-Rev	CTCCTGCTTGCGAAACTTGG
GATA3-For	TTCAGTTGGCCTAAGGTGGT
GATA3-Rev	CGCCGGACTCTTAGAAGCTA
TBX2-For	GGCCTTCCACAAGCTGAAG
TBX2-Rev	GCGGCTGGTACTTGTGCAT
ASCL1-For	GGAGCTTCTCGACTTCACCAA
ASCL1-Rev	GTTGTGCGATCACCCTGCT
MEIS1-For	TACCCGCACACAGCTCATAC
MEIS1-Rev	GGGAAGAGGGGGTGTCCATA
SOX4-For	GACCTGAACCCCAGCTCAAA
SOX4-Rev	AGCCGGGCTCGAAGTTAAAA
FN1-For	ACAAGCATGTCTCTCTGCCA
FN1-Rev	CCTCCAGAGCAAAGGGCTTA
ACTB-For	AGAGCTACGAGCTGCCTGAC
ACTB-Rev	AGCACTGTGTTGGCGTACAG
**sgRNA**	**Target sequence**
SOX4	GCTCCGCCTCTCGAATGAAA
MYC	GTATTTCTACTGCGACGAGG
NT1 (non-targeting)	GGGCCCGCATAGGATATCGC

Table S2. Oligo and primer sequences; Sequence information for primers used in quantitative RT-PCR and sgRNAs for CRISPR-mediated gene disruption.

Antibody	Assay	Manufacturer	Catalog #	Lot #
H3K27ac	ChIP-seq	Abcam	ab4729	GR3211959-1
H3K27me3	ChIP-seq	Abcam	ab192985	GR3204355-5
RARA	ChIP-seq	Abcam	ab41934	GR298513-2
MEIS1	ChIP-seq	Abcam	ab19867	GR153319-2
GATA3	CUT&RUN	Cell Signaling	5852	5
PHOX2B	CUT&RUN	Santa Cruz	sc-376993	E3012
SOX4	CUT&RUN	Sigma	HPA029901	D116454
MEIS1	CUT&RUN	Abcam	ab19867	GR153319-2
IgG	CUT&RUN	Proteintech	300000-0-AP	220000001
MYCN	Western blot	Cell Signaling	9045	2
MYC	Western blot	Cell Signaling	5605	8
Histone H3	Western blot	Cell Signaling	4499	9
HAND2	Western blot	Santa Cruz	sc-398167	K1716
ISL1	Western blot	DSHB	39.4D5	1-4-18
PHOX2B	Western blot	Santa Cruz	sc-376993	E3012
GATA3	Western blot	Cell Signaling	5852	5
ALK	Western blot	Cell Signaling	3333	11
TUBB3	Western blot	Cell Signaling	5568	7
ACTB	Western blot	Cell Signaling	4070	18
TBX2	Western blot	Santa Cruz	sc-514291	I2017
ASCL1	Western blot	Cell Signaling	43666	1
SOX4	Western blot	Santa Cruz	sc-518016	I1818
FN1	IF	Cell Signaling	26836	1
TUBB3	IF	Cell Signaling	5568	7
VIM	IF	Cell Signaling	5741	5
PCNA	IHC	EMD Millipore	MAB424R	3215515

Table S3. Antibody information: The antibodies used for ChIP-seq, Cut&Run, western blot, immunofluorescence (IF) and immunohistochemistry (IHC) are listed along with manufacturer, catalog number and specific lot numbers.

Cell line/Sample	Assay	Treatment	Target	GEO Accession #
BE2C	ChIP-seq	DMSO	H3K27ac	GSM4693011
BE2C	ChIP-seq	DMSO	H3K27me3	GSM4693012
BE2C	ChIP-seq	DMSO	RARA	GSM5210637
BE2C	ChIP-seq	DMSO	Input	GSM4693013
BE2C	ChIP-seq	ATRA, 5 µM, 12 d	H3K27ac	GSM4693006
BE2C	ChIP-seq	ATRA, 5 µM, 12 d	H3K27me3	GSM4693007
BE2C	ChIP-seq	ATRA, 5 µM, 12 d	Input	GSM4693009
BE2C	ChIP-seq	ATRA, 5 µM, 12 d	RARA	GSM4693005
BE2C	ChIP-seq	ATRA, 5 µM, 12 d	Input_RARA	GSM4693004
BE2C	ChIP-seq	ATRA, 5 µM, 12 d	MEIS1	GSM4693010
BE2C	ChIP-seq	ATRA, 5 µM, 12 d	Input_MEIS1	GSM4693008
NGP	ChIP-seq	DMSO	H3K27ac	GSM4693022
NGP	ChIP-seq	DMSO	Input	GSM4693023
NGP	ChIP-seq	ATRA, 5 µM, 12 d	H3K27ac	GSM4693018
NGP	ChIP-seq	ATRA, 5 µM, 12 d	RARA	GSM4693021
NGP	ChIP-seq	ATRA, 5 µM, 12 d	MEIS1	GSM4693020
NGP	ChIP-seq	ATRA, 5 µM, 12 d	Input	GSM4693019
NBL-S	ChIP-seq	DMSO	H3K27ac	GSM4693016
NBL-S	ChIP-seq	DMSO	Input	GSM4693017
NBL-S	ChIP-seq	ATRA, 5 µM, 12 d	H3K27ac	GSM4693014
NBL-S	ChIP-seq	ATRA, 5 µM, 12 d	Input	GSM4693015
BE2C	Cut & Run	DMSO	GATA3	GSM5211100
BE2C	Cut & Run	DMSO	PHOX2B	GSM5211103
BE2C	Cut & Run	DMSO	SOX4	GSM5211096
BE2C	Cut & Run	DMSO	MEIS1	GSM5211095
BE2C	Cut & Run	DMSO	IgG	GSM5211094
BE2C	Cut & Run	ATRA, 5 µM, 12 d	GATA3	GSM5211099
BE2C	Cut & Run	ATRA, 5 µM, 12 d	PHOX2B	GSM5211097
BE2C	Cut & Run	ATRA, 5 µM, 12 d	SOX4	GSM5211098
BE2C	Cut & Run	ATRA, 5 µM, 12 d	MEIS1	GSM5211101
BE2C	Cut & Run	ATRA, 5 µM, 12 d	IgG	GSM5211102
BE2C_rep1	RNA-seq	DMSO	N/A	GSM4693027
BE2C_rep2	RNA-seq	DMSO	N/A	GSM4693028
BE2C_rep3	RNA-seq	DMSO	N/A	GSM4693029
BE2C_rep1	RNA-seq	ATRA, 5 µM, 6 d	N/A	GSM4693024
BE2C_rep2	RNA-seq	ATRA, 5 µM, 6 d	N/A	GSM4693025
BE2C_rep3	RNA-seq	ATRA, 5 µM, 6 d	N/A	GSM4693026
NGP_rep1	RNA-seq	DMSO	N/A	GSM4693039
NGP_rep2	RNA-seq	DMSO	N/A	GSM4693040
NGP_rep3	RNA-seq	DMSO	N/A	GSM4693041
NGP_rep1	RNA-seq	ATRA, 5 µM, 6 d	N/A	GSM4693036
NGP_rep2	RNA-seq	ATRA, 5 µM, 6 d	N/A	GSM4693037
NGP_rep3	RNA-seq	ATRA, 5 µM, 6 d	N/A	GSM4693038
NBL-S_rep1	RNA-seq	DMSO	N/A	GSM4693033
NBL-S _rep2	RNA-seq	DMSO	N/A	GSM4693034
NBL-S _rep3	RNA-seq	DMSO	N/A	GSM4693035
NBL-S _rep1	RNA-seq	ATRA, 5 µM, 6 d	N/A	GSM4693030
NBL-S _rep2	RNA-seq	ATRA, 5 µM, 6 d	N/A	GSM4693031
NBL-S _rep3	RNA-seq	ATRA, 5 µM, 6 d	N/A	GSM4693032

Table S4. NCBI GEO accession numbers; raw and processed data files are deposited to the NCBI GEO server under super-series GSE155002.
